# Characterization of the complete mitochondrial genome of *Garra motuoensis* (Cypriniformes: Cyprinidae) and its phylogenetic position within genus *Garra*

**DOI:** 10.1080/23802359.2022.2064247

**Published:** 2022-04-19

**Authors:** Zheng Gong, Xiaobing Li, Dayong Chang, Henglun Shen

**Affiliations:** a College of Life Sciences, Zaozhuang University, Zaozhuang, China; b The Key Laboratory of Aquatic Biodiversity and Conservation, Institute of Hydrobiology, Chinese Academy of Sciences, Wuhan, China; c College of Fisheries, Southwest University, Chongqing, China

**Keywords:** *Garra motuoensis*, mitochondrial genome, phylogenetic analysis

## Abstract

*Garra motuoensis*, an endemic labeonine fish, was reported distributed in the lower Yarlung Tsangpo River drainage with little published biological information. Herein, we sequenced and characterized the complete mitochondrial genome of *G. motuoensis*, which was 16,806 bp in length, containing 13 PCGs, 22 tRNA genes, two rRNA genes, one light strand replication origin (*O_L_*), one control region (D-loop), and one replication region. Phylogenetic analysis based on 13 PCGs sequences revealed that *G. motuoensis* had a closest relationship with *G. qiaojiensis*. Then, both species clustered with other species of *Garra*, and next grouped with other genera of subfamily Labeoninae.

The genus *Garra* Hamilton 1822, the largest genus in the subfamily Labeoninae of family Cyprinidae, is a group of small to middle-sized benthic freshwater fishes widespread across tropical and sub-tropical waters from South China eastwards to West Africa westwards (Menon 1964). Species of this genus primarily inhabiting swift-flowing rivers and mountain streams, which are distinguished by the slender and subcylindrical body, crenulated rostral fold on the upper lip, and specialized mental adhesive disc on the lower lip (Chen et al. 2009; Nebeshwar and Vishwanath 2017).

In the Motuo reach of the lower Yarlung Tsangpo River drainage, there was only one *Garra* species ever recorded, i.e. *G. kempi* (Zhang et al. 1995; Yue 2000). However, based on recent ichthyological surveys, the original recorded *G. kempi* was revised as a new species *G. tibetana* (Gong et al. 2018a). Meanwhile, another two new species (*G. motuoensis* and *G. yajiangensis*) of this genus were described from this area (Gong et al. 2018b). As an endemic fish in this drainage, *G. motuoensis* was known only distributed in the Xigong River, a tributary of the Yarlung Tsangpo River. To date, studies on this species were quite limited except its taxonomic description.

In this study, specimen of *G. motuoensis* was collected from its type locality, the Xigong River (29°16′44″N, 95°14′56″E, 680 m elevation), which was deposited at the Museum of Aquatic Organisms at the Institute of Hydrobiology (IHB), Chinese Academy of Sciences under the voucher no. IHB 20161473 (contact person: Dr Huanshan Wang, hswang@ihb.ac.cn). The genomic DNA was extracted from muscle tissue using the protocol of Genomic DNA Extraction Kit (Tsingke, Beijing, China) according to the manufacturer's instruction. The genomic DNA sequences were determined through Illumina NovaSeq platform (Illumina, San Diego, CA). The mitochondrial genome was assembled using SPAdes 3.9 under the 150 paired-end reading strategy (Bankevich et al. 2012) with *G. qiaojiensis* as the reference species (Xiong et al. 2016). Then, all obtained genes were annotated in MITOS Web Server (Bernt et al. 2013).

Results showed that the complete mitogenome of *G. motuoensis* was a circular molecular with the length of 16,806 bp (GenBank accession no. OK375462) and contained 13 protein-coding genes (PCGs), 22 transfer RNA (tRNA) genes, two ribosomal RNA (rRNA) genes, and three non-coding regions: one light strand replication origin (*O_L_*), one control regions (D-loop), and one replication region. Among these genes, nine genes (*tRNA^Gln^*, *tRNA^Ala^*, *tRNA^Asn^*, *tRNA^Cys^*, *tRNA^Tyr^*, *tRNA^Ser^*, *ND6*, *tRNA^Glu^*, and *tRNA^Pro^*) were encoded on the minority strand, while the remaining were located at the majority strand. Most PCGs used ATN as the initiation codon except *COX1* started with GTG. In addition to *COX2* and *ND4* with an incomplete termination codon ‘T’, the rest were encoded by the typical termination codons ‘TAR’. The lengths of single non-coding regions for *O_L_*, D-Loop and replication region were 35 bp, 876 bp, and 273 bp, respectively.

The nucleotide sequences of 13 PCGs (11,367 bp) were combined from *G. motuoensis* and 21 additional species including 10 species of *Garra*, 10 species of other labeonine genera and one outgroup (*Neolissochilus hexagonolepis*) for phylogenetic analysis. Sequence alignment was performed using MEGA 6.0 (Kumar et al. 2018). Nucleotide substitution model (GTR + I+G) was selected based on corrected Akaike information criterion (AICc) using PartitionFinder 2.1 in PhyloSuite 1.2 platform (Zhang et al. 2020). The phylogenetic tree was constructed based on Bayesian inference method using MrBayes 3.1 (Ronquist et al. 2012).

Topology of the phylogenetic tree indicated that species of genus *Garra* formed a highly supported monophyletic group in the subfamily Labeoninae. Specifically, *G. motuoensis* was a sister clade to *G. qiaojiensis* with the Kimura 2-parameter distance of 10.6%. Then, both species clustered with other congeners of *Garra*, and next grouped with species of other labeonine genera (Figure 1). The present study enriched the genetic database of genus *Garra*, and also expanded our understanding of the phylogenetic relationships within the subfamily Labeoninae.

**Figure 1.**
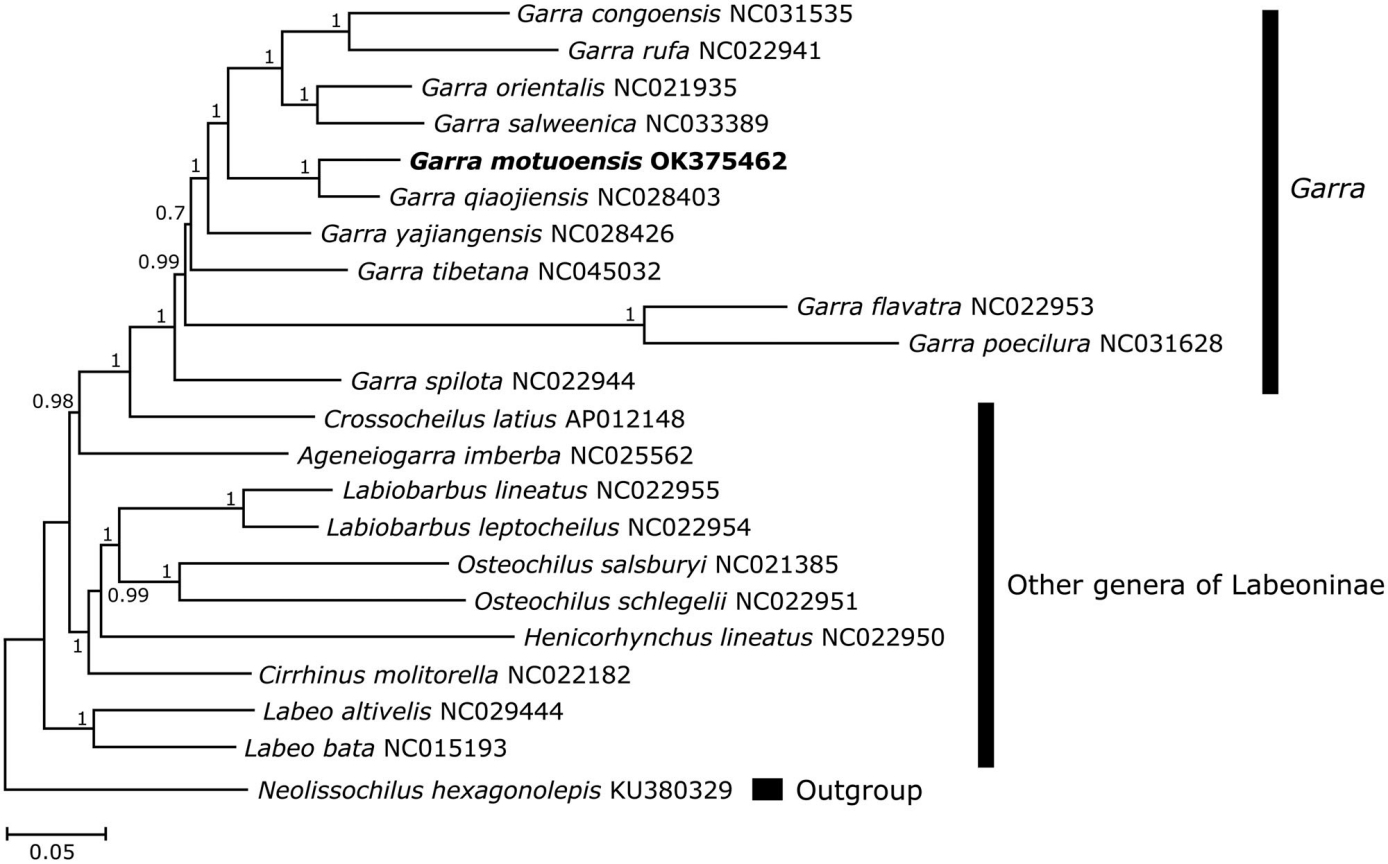
Phylogenetic tree of 21 labeonine fishes derived from Bayesian inference based on the combined nucleotide sequences of 13 protein-coding genes. The values on the nodes indicated the posterior probabilities. The GenBank accession numbers of included species were shown behind the taxon names.

## Data Availability

The data that support the findings of this study are openly available in GenBank of NCBI at https://www.ncbi.nlm.nih.gov, under the accession no. OK375462. The associated BioProject, SRA, and Bio-Sample numbers are PRJNA785075, SRR17110448, and SAMN23526806, respectively.
